# Research on the Molecular Mechanisms and Key Gene Discovery in *Quercus variabilis* Root Pruning Based on Transcriptomics and Hormone Profiling

**DOI:** 10.3390/ijms252111541

**Published:** 2024-10-27

**Authors:** Hao Dou, Jiajia Sun, Xi Feng, Huyang Lyu, Zhen Qin, Ruoyi Ni, Yilin Wang, Huijuan Sun, Xin Zhou, Wu Tang, Jin’e Quan, Xitian Yang

**Affiliations:** College of Forestry, Henan Agricultural University, Zhengzhou 450002, China; douhao@stu.henau.edu.cn (H.D.); dearperi@163.com (J.S.); fengxi@stu.henau.edu.cn (X.F.); lvhuyang@stu.henau.edu.cn (H.L.); 15670228779@163.com (Z.Q.); niruoyi1997@163.com (R.N.); wangtian@stu.henau.edu.cn (Y.W.); sunmoon0415@163.com (H.S.); zhouxin@stu.henau.edu.cn (X.Z.); tangwu@stu.henau.edu.cn (W.T.)

**Keywords:** *Quercus variabilis*, plant hormone, hub genes, hormone profiling

## Abstract

*Quercus variabilis* (*Q. variabilis*), a significant broadleaf species used in afforestation across high, sandy, and mountainous regions, presents unique challenges for transplantation. This species is characterized by its slow above-ground growth and rapid taproot development, which suppresses the proliferation of lateral and fibrous roots, negatively impacting post-transplant survival. Research indicates that targeted root pruning—specifically, the removal of one-third of the roots—promotes the development of lateral roots in these seedlings. This study involved pruning the root systems of *Q. variabilis* and assessing the subsequent root development in comparison to an unpruned control group. Our analysis, which included transcriptome sequencing and plant hormone metabolism assays conducted at 2, 12, and 25 days post-pruning, yielded 126.02 Gb of clean data and identified 7662 differentially expressed genes (DEGs). These genes were primarily enriched in the plant hormone signal transduction pathway. Further investigation of this pathway, along with hormone content measurements, elucidated the mechanisms that contribute to enhanced root growth following pruning. Additionally, through a weighted correlation network analysis (WGCNA), we identified 20 key genes that are instrumental in promoting root development in *Q. variabilis* saplings. This research advances the theoretical framework for forestry seedling production and afforestation, laying the groundwork for scientifically informed vegetation restoration techniques.

## 1. Introduction

The ecological functions of forests are largely driven by the actions of plant root systems. Enhancing the survival rate of seedlings is essential for improving their quality [1]. In the contexts of transplantation and transportation, root pruning is often deemed necessary. However, excessive pruning can damage root tissues, adversely affecting both nutrient utilization and the growth and development of the seedlings [2]. A well-structured root system not only facilitates nutrient absorption and supports the physiological functions of plants but also helps seedlings adapt to complex environments [3,4]. Root pruning, commonly applied to container-grown seedlings, typically involves severing the main root during the nursery phase before transplantation into containers or fields [5]. This technique provides several advantages, including the conservation of seed resources, standardization of growth, simplification of management, and an increase in the rate at which seedlings are ready to leave the nursery [6]. Research, such as the studies conducted by Du Yangwen on grafted thin-shell walnut seedlings, has shown that an optimal root-cutting ratio can enhance characteristics such as root length, diameter, and total dry weight, thereby improving the quality indices of seedlings [7]. This research has also explored the effects of various forms of root pruning by examining characteristics like root surface area, root length, root volume, number of roots, and root diameter [8]. Root pruning significantly influences the morphological formation of seedling roots, characterized by the suppression of main root growth and the stimulation of increased lateral roots, which result in a dense and well-developed fibrous root system [9]. Studies have shown that in Oriental arborvitae subjected to root pruning, both the number of lateral roots and the total root mass are greater than those observed in unpruned seedlings, with the lateral roots typically developing closer to the root base than in unpruned seedlings [10]. To date, various plants, such as *Pinus massoniana* Lamb [11], *Pinus elliottii* [12], *Castanopsis fargesii* [13], and *Q. variabilis* [14], have been extensively utilized in seedling root-pruning practices. In conclusion, to enhance afforestation efficiency, it is crucial that lateral roots are evenly distributed within the soil to ensure the stability of the plant root system. Furthermore, the growth and development of plant root systems are influenced by factors such as plant hormones, environmental influences, and genetic elements, which respond to environmental changes through these interactions [15].

Root system formation in plants is a developmental process regulated by hormonal and environmental factors. Extensive research has demonstrated that low levels of cytokinin (CTK) combined with high levels of auxin (IAA) in the root system promote the differentiation and formation of adventitious roots [16,17]. During hormone-induced root development, the balance between IAA and CTK significantly influences root cell division [18]. Elevated levels of CTK are known to encourage bud formation but inhibit root development [19]. At the onset of lateral root development, abscisic acid (ABA) acts not only as an antagonist to auxins but also plays a crucial role in regulating the balance between auxins and CTK, indirectly controlling the formation of lateral roots [20,21]. Studies by Wang Xiaoling et al. [22] have shown that ABA can mitigate the inhibitory effects of high concentrations of IAA on root growth in tetraploid black locust, thereby facilitating successful rooting in cuttings. Additionally, the influence of strigolactones on root growth may be mediated by auxin efflux carriers [23]. Plant endogenous hormones intricately regulate plant growth through the concentration and balance of individual hormones.

*Q. variabilis*, a tall deciduous tree from the genus Quercus within the Fagaceae family, can reach up to 30 m in height. It features bark with deep vertical fissures, glabrous twigs, and leaves that are either ovate–lanceolate or elongated elliptical–lanceolate with broadly cuneate to nearly rounded bases and spiny serrations. The acorn cup is cup-shaped with a flat top. *Q. variabilis* flowers from March to April and bears fruit from September to October of the following year [24]. Native to the Chinese provinces of Liaoning, Hebei, Shanxi, and Shaanxi, *Q. variabilis* is both light-demanding and shade-tolerant, capable of withstanding cold and drought conditions, and thrives in nutrient-poor soils [25,26]. The fruit of *Q. variabilis* is utilized for its antitussive properties, to treat tinea capitis, and as a diuretic [27,28]. It is also used in garden landscaping and is a primary species for afforestation in China’s sandy, mountainous regions. The growth characteristics of *Q. variabilis* seedlings include slow above-ground development and rapid taproot expansion, which leads to a reduction in lateral and fibrous roots, significantly affecting transplant survival [14]. To address this, root pruning is employed to inhibit main root growth while promoting the proliferation of lateral roots, creating a dense and well-developed fibrous root system. This technique improves afforestation success by ensuring an even distribution of lateral roots within the soil and providing deep anchorage, essential for plant stability [29,30,31]. Our research team has observed that pruning one-third of the roots enhances the survival and physiological condition of *Q. variabilis* compared to unpruned specimens, although the precise molecular mechanisms involved remain to be fully elucidated [32].

This study focuses on *Q. variabilis*, commonly used in afforestation, and examines the effects of one-third root pruning on root growth, physiological traits, and biomass distribution. Supported by transcriptomic and hormone group analyses, the research identifies hormones that are differentially expressed in the root systems of pruned *Q. variabilis* saplings. Using bioinformatics, it also identifies the key genes induced by root pruning that are critical for the development of the root system in these saplings, thus exploring how pruning influences the molecular mechanisms regulating root system development in *Q. variabilis*.

## 2. Results

### 2.1. Dynamic Changes in Root Morphology of Q. variabilis Saplings After Root Pruning

**Figure 1:**
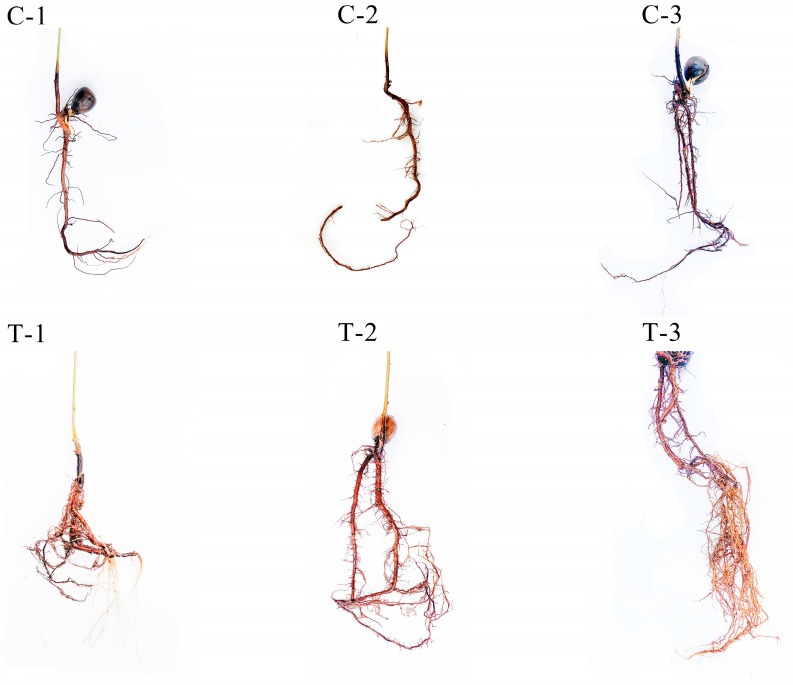
Dynamic changes in root morphology of *Q. variabilis* saplings in control and treatment groups.

The main roots of the *Q. variabilis* saplings were pruned to one-third of their original length, with unpruned saplings serving as controls. Morphological assessments of new lateral root formation in *Q. variabilis* saplings are depicted in Figure 1. At 2 days post-pruning, there were no significant differences in root characteristics between the control and treatment groups. By day 12, an increase in the length of the main roots was observed in both groups, with the treatment group exhibiting numerous new milky white lateral roots at the pruning site. By day 25, significant increases in root length, volume, and surface area were recorded in both groups, with the treatment group showing superior growth metrics compared to the control.

### 2.2. Effects of Root Pruning on the Root Morphological and Physiological Indicators of Q. variabilis Saplings

**Figure 2:**
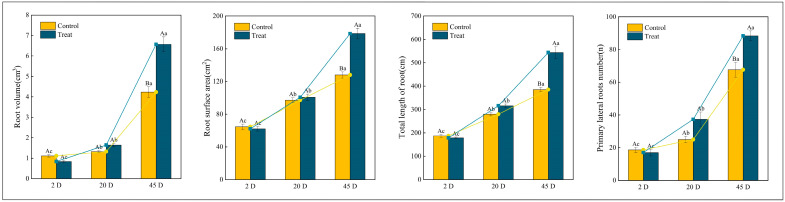
Changes in root morphological indicators of *Q. variabilis* saplings in control and treatment groups. Different uppercase letters indicate significant differences between treatments within the same time period (*p* < 0.05), while the same uppercase letters denote no significant difference (*p* > 0.05). Different lowercase letters signify significant differences within the same treatment across different time periods (*p* < 0.05), and the same lowercase letters indicate no significant differences (*p* > 0.05).

Figure 2 illustrates the morphological indicators of *Q. variabilis* saplings’ roots before and after root pruning, observed over three distinct time intervals. At 2 days, the total root length and the number of first-order lateral roots in the control group were marginally greater than those in the treatment group, although these differences were not statistically significant (*p* > 0.05). By day 12, there was a statistically significant increase in the number of first-order lateral roots in the treatment group compared to the control group (*p* < 0.05). By day 25, both groups exhibited significant increases in total root length and number of lateral roots compared to earlier assessments. Notably, the treatment group demonstrated a total root length and number of lateral roots that were 1.14 and 1.32 times those of the control group, respectively. While the root surface area and volume were comparable between the two groups at 2 days and 12 days, by day 25, these metrics in the treatment group were 1.39 and 2.04 times those of the control group, respectively. These findings suggest that *Q. variabilis* saplings adjust their root morphology in response to root pruning, resulting in increased total root length, root surface area, root volume, and number of first-order lateral roots, which collectively contribute to the development of a more robust root system structure.

### 2.3. Transcriptome Sequencing Data Analysis

#### 2.3.1. Analysis of Transcriptome Output Data and Assembly of Transcripts

**Figure 3:**
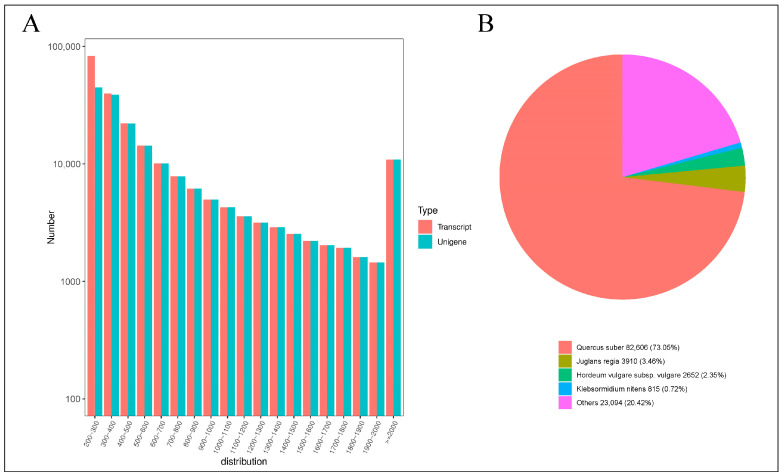
Statistical analysis of transcripts. (**A**) Distribution graph of sequence lengths (**B**) NR annotation pie chart.

Utilizing the Illumina HiSeq high-throughput sequencing platform, 18 cDNA libraries were processed, yielding a collective total of 126.02 Gb of clean data. Each sample generated approximately 6 Gb of clean data, with a Q30 base percentage exceeding 90%, indicating a high level of sequencing-data quality (Table S2). Subsequently, the clean reads were assembled and clustered. The results of this assembly, as documented in Table S3, yielded 185,621 Unigene sequences and 224,579 Transcript sequences. The distribution of sequence lengths is depicted in Figure 3A. The assembled Unigene sequences were aligned against several databases, including KEGG, NR, Swiss-Prot, GO, COG/KOG, Trembl, and Pfam. The annotation results for these Unigenes are detailed in Table S4. Specifically, the numbers of Unigenes annotated in the KEGG, NR, Swiss-Prot, Trembl, KOG, GO, and Pfam databases were 80,074; 113,077; 70,594; 99,758; 65,415; 84,286; and 75,442, respectively. Notably, a significant 60.92% of the Unigenes were annotated in the NR database, which serves as a primary resource for functional annotation, providing robust support for the functional analysis of *Q. variabilis* sequences. The results of querying the NR database are presented in Figure 3B. This query revealed that the species most closely matching the sequences from *Q. variabilis* is *Quercus suber*, which constitutes 73.05% of the matches. Both *Q. variabilis* and *Quercus suber* belong to the genus Quercus within the Fagaceae family. This high level of sequence similarity supports the accuracy of the assembly and annotation processes applied to the *Q. variabilis* transcript sequences in this study.

#### 2.3.2. Alignment and Reproducibility Test of Transcript

After assembly, the transcripts were clustered, and the deduplicated transcripts were designated as reference sequences. Subsequently, clean reads from each sample were aligned to these reference sequences, achieving an alignment efficiency ranging from 80.79% to 84.29% across the 18 samples, indicative of high-quality sequencing data (Table S5).

To assess the reproducibility of biological replicates, a correlation analysis was performed using Pearson’s Correlation Coefficient (r) as the metric. This analysis, depicted in Figure S1, demonstrated a strong correlation in gene expression levels among biological replicates within the same treatment, though some variability was observed across different treatments. These results substantiate the high reliability and robustness of the experimental data, confirming its suitability for subsequent detailed analyses.

#### 2.3.3. Identification and Analysis of DEGs

**Figure 4:**
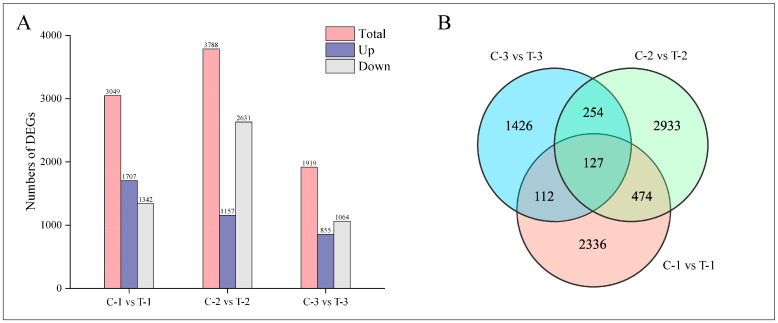
Distribution of DEGs at different time periods for control and treatment groups. (**A**) Bar chart displaying the number of DEGs at various time intervals. (**B**) Venn diagram illustrating the intersections of DEGs across different time periods.

Normalization of the mapped reads and transcript lengths was conducted using FPKM to quantify transcript or gene expression levels. The criteria for selecting DEGs included an absolute log2-fold change of at least 1 and an FDR below 0.05. This analysis identified a total of 7662 DEGs across three time intervals following root pruning, as illustrated in Figure 4A. Specifically, at 2 days post-pruning, the treatment group exhibited 3049 DEGs compared to the control group, including 1707 upregulated and 1342 downregulated genes; at 12 days, there were 3788 DEGs, with 1157 upregulated and 2631 downregulated genes; and at 25 days, 1919 DEGs were identified, comprising 855 upregulated and 1064 downregulated genes. Figure 4B presents a quantitative analysis of these DEGs, highlighting 601 common DEGs between 2 days and 12 days; 239 between 2 days and 25 days; 381 between 12 days and 25 days; and 127 DEGs common across all three time periods.

#### 2.3.4. GO Enrichment Analysis of DEGs

**Figure 5:**
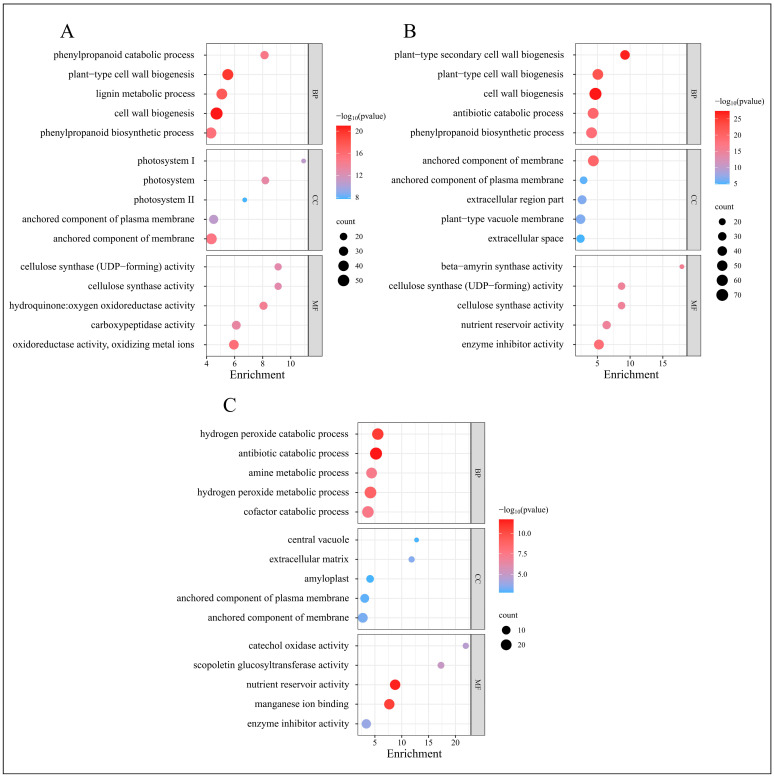
GO enrichment analysis of DEGs from four time periods. (**A**) GO enrichment results of EDGs in C-1 vs. T-1. (**B**) GO enrichment results of EDGs in C-2 vs. T-2. (**C**) GO enrichment results of EDGs in C-3 vs. T-3.

GO classifies genes into three categories: Biological Process, Molecular Function, and Cellular Component. We conducted a GO enrichment analysis on the DEGs, selecting the five GO terms with the lowest *p*-values from each category to generate GO enrichment bubble charts, as displayed in Figure 5.

At 2 days, the GO enrichment results for the DEGs in the treatment group, compared to the control group, are depicted in Figure 5A. The most enriched GO terms within the Biological Process category include “cell wall biogenesis”, “plant-type cell wall biogenesis”, and “lignin metabolic process”. In the Cellular Component category, the predominant terms are “anchored component of membrane”, “photosystem”, and “photosystem I”. The Molecular Function category features enriched terms such as “oxidoreductase activity, oxidizing metal ions”, “hydroquinone oxidoreductase activity”, and “carboxypeptidase activity”.

At 12 days, as shown in Figure 5B, the most enriched GO terms in the Biological Process category include “cell wall biogenesis”, “plant-type secondary cell wall biogenesis”, and “plant-type cell wall biogenesis”. The Cellular Component category highlights terms such as “anchored component of membrane”, “extracellular region part”, and “plant-type vacuole membrane”. For Molecular Function, the enriched terms include “enzyme inhibitor activity”, “beta-amyrin synthase activity”, and “cellulose synthase activity”.

At 25 days, Figure 5C illustrates the GO enrichment results, with the Biological Process category featuring terms such as “antibiotic catabolic process”, “hydrogen peroxide catabolic process”, and “hydrogen peroxide metabolic process”. The Cellular Component category includes “extracellular matrix”, “anchored component of membrane”, and “anchored component of plasma membrane”. The Molecular Function category is characterized by “nutrient reservoir activity”, “manganese ion binding”, and “scopoletin glucosyltransferase activity”.

The GO enrichment analysis reveals significant biological processes across different phases post-root-pruning: extensive synthesis of root cell walls, enhanced lignin metabolism, and robust redox reactions during the initial phase; synthesis of vacuoles and cellulose, alongside intensified enzyme-catalyzed reactions during the differentiation phase; and active hydrogen peroxide metabolism and nutrient interactions during the elongation phase. These findings elucidate the molecular metabolic processes following root pruning, potentially underlying the observed increase in root proliferation.

#### 2.3.5. KEGG Enrichment Analysis of DEGs

**Figure 6:**
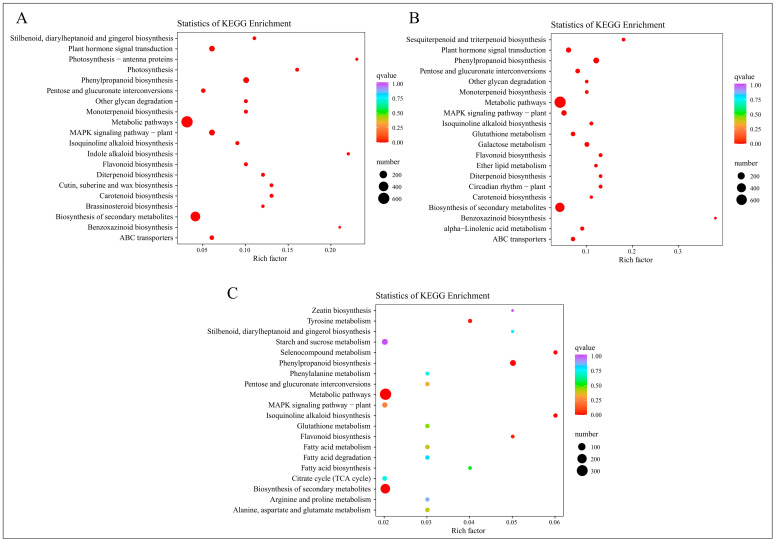
KEGG enrichment analysis of DEGs from four time periods. (**A**) KEGG enrichment results of EDGs in C-1 vs. T-1. (**B**) KEGG enrichment results of EDGs in C-2 vs. T-2. (**C**) KEGG enrichment results of EDGs in C-3 vs. T-3.

Following the annotation of differentially expressed genes to KEGG pathways, a KEGG enrichment analysis was executed. The 20 KEGG pathways with the lowest Q-values were selected for representation in a KEGG enrichment scatter plot, as illustrated in Figure 6.

At 2 days, the DEGs are prominently enriched in pathways such as “Stilbenoid, diarylheptanoid and gingerol biosynthesis”, “Plant hormone signal transduction”, “Photosynthesis-antenna proteins”, and “Photosynthesis”, as depicted in Figure 6A. At 12 days, Figure 6B shows significant enrichment in pathways including “Sesquiterpenoid and triterpenoid biosynthesis”, “Plant hormone signal transduction”, “Phenylpropanoid biosynthesis”, and “Pentose and glucuronate interconversions”. At 25 days, the pathways predominantly enriched are “Zeatin biosynthesis”, “Tyrosine metabolism”, “Stilbenoid, diarylheptanoid and gingerol biosynthesis”, and “Starch and sucrose metabolism”, as indicated in Figure 6C.

In summary, the KEGG enrichment results signify heightened metabolic activity post-root-pruning, with accelerated synthesis of compounds such as plant hormones, stilbenoids, diarylheptanoids, sesquiterpenoids, and triterpenoids. Notably, pathways related to plant hormones are significantly enriched across all studied time periods, with “Plant hormone signal transduction” prominent in the first two periods and “Zeatin biosynthesis” at 25 days, all exhibiting the lowest Q-values. This suggests that plant hormones play a pivotal role following root pruning, aligning with prior research and highlighting their potential importance in understanding root system development post-pruning.

### 2.4. Hormone Data Analysis

#### 2.4.1. Quality Control and Sample-Clustering Analysis of Hormone Profiling

As illustrated in Figure S2, the total ion chromatograms (TICs) from the quality-control samples showed considerable overlap in retention times and peak intensities, indicating stable instrument performance throughout the study. The hormone data were normalized, and a heatmap analysis was conducted on all samples, with the results depicted in Figure S3. The clustering outcomes for each treatment group were consistent, confirming the reliability of the hormone data for subsequent analyses.

#### 2.4.2. Differential Hormones Screening and Counting

**Figure 7:**
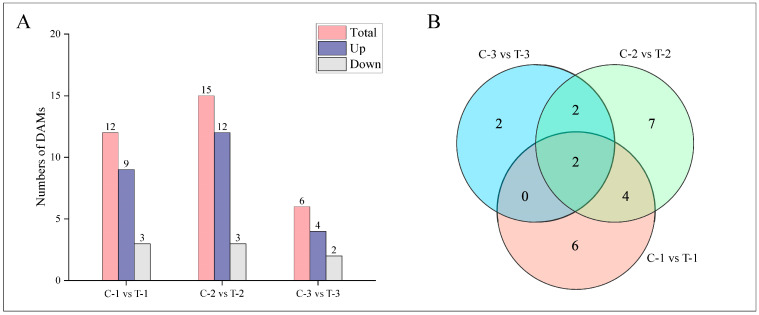
Distribution of DAMs at different time periods for control and treatment groups. (**A**) Bar chart depicting the number of DAMs at various time points. (**B**) Venn diagram illustrating the intersections of DAMs at different periods.

Metabolites were analyzed for significant differences using the criteria of Fold_Change ≥ 2, Fold_Change ≤ 0.5, and *p*-value ≤ 0.01, as shown in Figure 7A. Two days post-treatment, the treatment group exhibited a total of 12 differentially accumulated metabolites (DAMs), with 9 upregulated and 3 downregulated. By day 12, there were 15 DAMs, with 12 upregulated and 3 downregulated. At 25 days, the analysis identified 6 DAMs, with 4 upregulated and 2 downregulated. The correlations among groups are detailed in Figure 7B. At 2 days and 12 days, six DAMs were common, including ABA, JA, cZ, DHZ7G, K, and JA-Val. Between 2 days and 25 days, two DAMs were consistent: ABA and K. Between 12 days and 25 days, four DAMs were observed consistently: ABA, BAP, K, and JA-ILE. ABA and K were the three DAMs shared across all periods, suggesting their continual activity and potential role in enhancing lateral root formation compared to the control group.

#### 2.4.3. KEGG Enrichment Analysis of Differential Hormones

**Figure 8:**
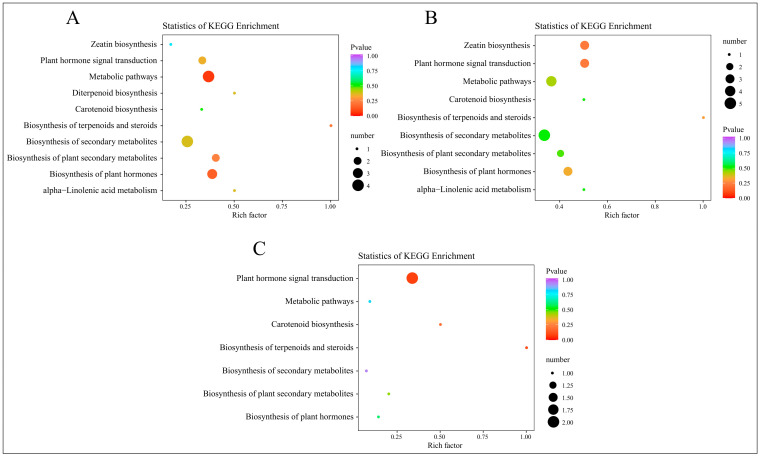
KEGG enrichment analysis of DAMs from four time periods. (**A**) KEGG enrichment results of DAMs in C-1 vs. T-1. (**B**) KEGG enrichment results of DAMs in C-2 vs. T-2. (**C**) KEGG enrichment results of DAMs in C-3 vs. T-3.

After mapping the differential hormones to KEGG pathways, we conducted an enrichment analysis using a hypergeometric test. The results were visualized in a scatter plot of KEGG enrichment, arranged by ascending *p*-values, as depicted in Figure 8.

At 2 days, the KEGG enrichment analysis for the DAMs in the treatment group, compared to the control group, is displayed in Figure 8A. This analysis highlights significantly enriched pathways, including zeatin biosynthesis, plant hormone signal transduction, metabolic pathways, and diterpenoid biosynthesis. By 12 days, as shown in Figure 8B, the analysis indicates enrichment in similar pathways, adding carotenoid biosynthesis. At 25 days, the results in Figure 8C show significant enrichment in pathways such as plant hormone signal transduction, metabolic pathways, carotenoid biosynthesis, and the biosynthesis of terpenoids and steroids. These findings underscore the integral role of these metabolites in plant hormone pathways and processes such as carotenoid biosynthesis. Considering the combined results from both transcriptomic and hormone profiling in the KEGG analysis, it is clear that plant hormone signal transduction is significantly enriched following root pruning. This enrichment underscores the importance of this pathway as a critical area for investigating the mechanisms behind enhanced root formation.

### 2.5. Plant-Hormone-Signaling Pathway Was Affected by Root Pruning

**Figure 9:**
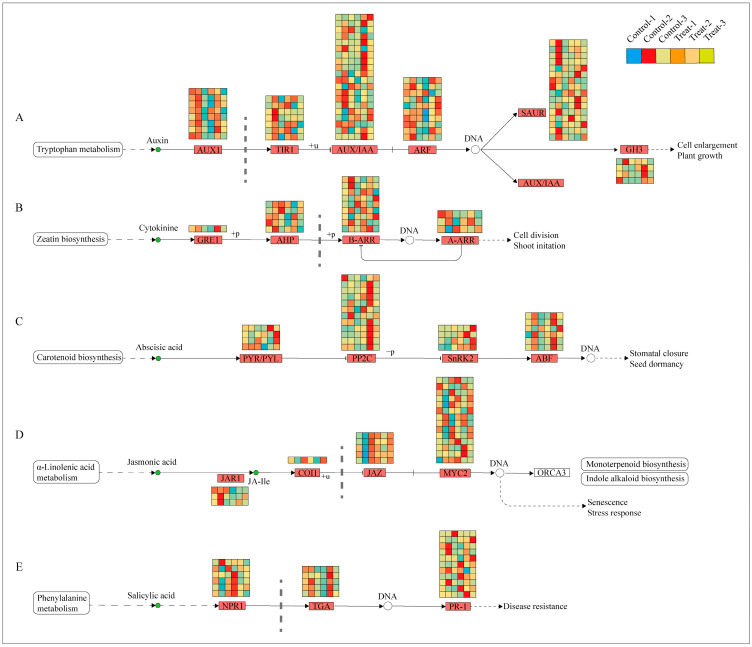
Expression of DEGs in plant hormone pathways. (**A**) Represents the IAA signal transduction pathway; (**B**) represents the CTK signal transduction pathway; (**C**) represents the ABA signal transduction pathway; (**D**) represents the JA signal transduction pathway; (**E**) represents the SA signal transduction pathway.

Previous Sections have demonstrated that the plant hormone signal transduction pathway was the most active across the examined physiological processes. Consequently, a combined transcriptomic and metabolomic analysis was undertaken to delve deeper into this pathway, revealing significant variations in five primary hormones: IAA, CTK, JA, salicylic acid (SA), and ABA. The biosynthesis pathways of these hormones were analyzed, with the results presented in Figure 9. In the auxin biosynthesis pathway, 65 differentially expressed genes were identified, with the largest subset (20 genes) belonging to the AUX/IAA family. These genes were predominantly expressed at 12 days, suggesting that auxin’s influence is most pronounced during this period. The marked difference in expression between the treatment and control groups at 12 days suggests that this time-point is critical for observing differential responses to treatment. In the cytokinin biosynthesis pathway, 18 differentially expressed genes were noted, with the highest number (9 genes) in the B-ARR family, also showing high expression at 12 days. The pathways for ABA, JA, and SA enriched 26, 23, and 22 genes, respectively; however, their expression differences were not as concentrated at 12 days, instead exhibiting variability across different periods.

**Figure 10:**
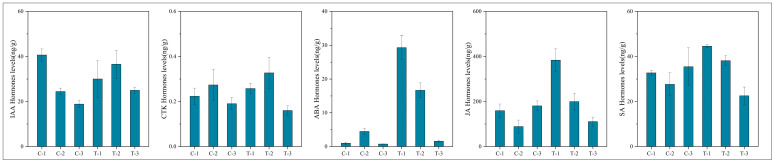
Graphs showing the content of five plant hormones at various periods.

**Figure 11:**
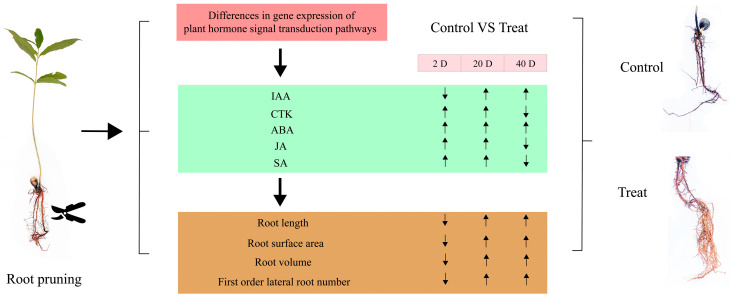
Diagram of the series of physiological activities occurring after root pruning treatment. The green square indicates the trend of hormone change, the yellow square indicates the trend of root physiological state change, and the arrow indicates up-regulated or down-regulated.

To assess the significance of each period in the two treatments, the content of plant hormone metabolomes was tested at each respective period. The results, depicted in Figure 10, indicate that compared to the control group, the treatment group’s auxin content displayed a pattern of decrease, then increase, and finally increase at 2 days, 12 days, and 25 days, respectively. CTK, JA, and SA showed a pattern of increase, increase, and decrease, respectively, across these periods in the treatment group. In contrast, ABA exhibited an increasing trend in the treatment group across all three periods. These variations in hormone levels suggest that root pruning leads to changes in root hormone content, with the four hormones promoting root development (IAA, CTK, JA, and SA) mitigating the effects of ABA, which is less favorable for root development. This results in enhanced root formation in the treatment group. The physiological activities post-pruning were synthesized by integrating physiological status with transcriptomic and hormone profiling data, as illustrated in Figure 11. Variations in gene expression induced changes in plant hormone content, ultimately leading to different physiological states.

### 2.6. Weighted Gene Co-Expression Network Analysis

#### 2.6.1. Selection of Soft Threshold, Module Hierarchical Clustering, and Key Module Selection

**Figure 12:**
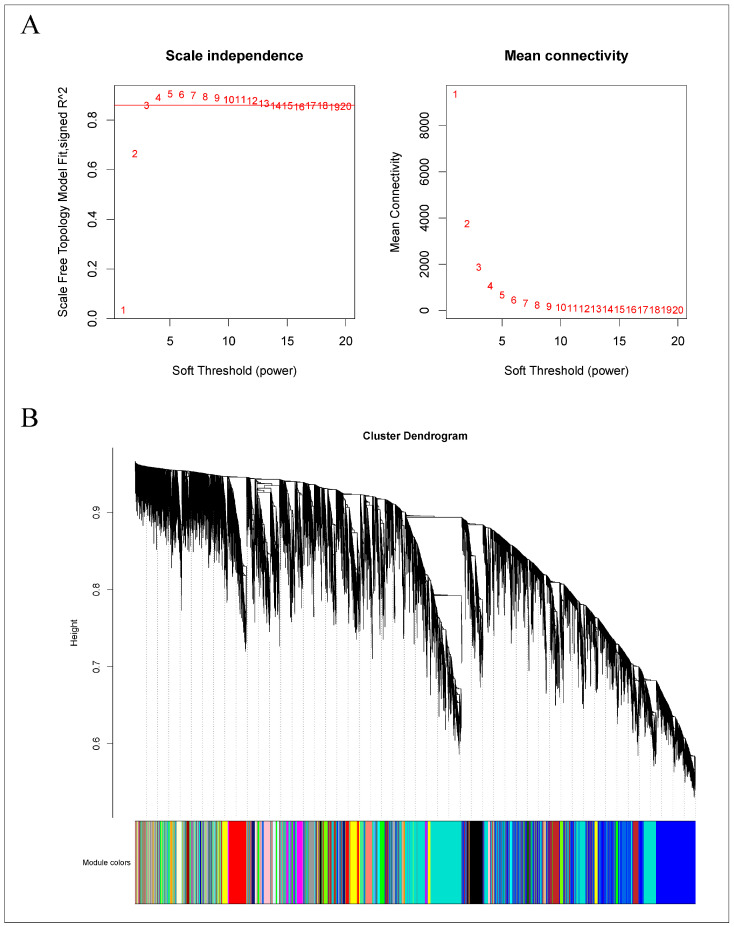
WGCNA of the gene expression matrix in *Q. variabilis*. (**A**) The most appropriate soft threshold was determined by plotting scale independence and mean connectivity. (**B**) A dendrogram based on co-expression network analysis depicts the hierarchical clustering of genes, with the module colors represented on the X-axis. Each color represents a module.

As demonstrated in Figure 12A, a power value of 13 achieved a scale-free network fit index (R2) exceeding 0.85, with the average connectivity approaching zero, confirming the establishment of a scale-free topology as required. Subsequently, modules were identified using a dynamic tree-cut method, with modules exhibiting more than 75% similarity being merged. This process resulted in the formation of 29 co-expression modules, each represented by a distinct color, as shown in Figure 12B. The gene count within each module varied; the turquoise module contained the most genes, numbering 1223, while the saddle-brown module had the fewest, with 77 genes. Other modules contained gene counts ranging from 77 to 1223. The gray module includes genes that were not assigned to any other module.

A subsequent correlation analysis between these 30 modules and the samples, presented in Figure S4, showed that the orange and light-yellow modules exhibited a high degree of correspondence with the treatment group. It is postulated that the genes within the orange (152 genes) and light-yellow (305 genes) modules demonstrate specificity post-root-pruning; hence, these two modules were selected for focused analysis.

#### 2.6.2. Selection and Functional Analysis of Hub Genes

**Figure 13:**
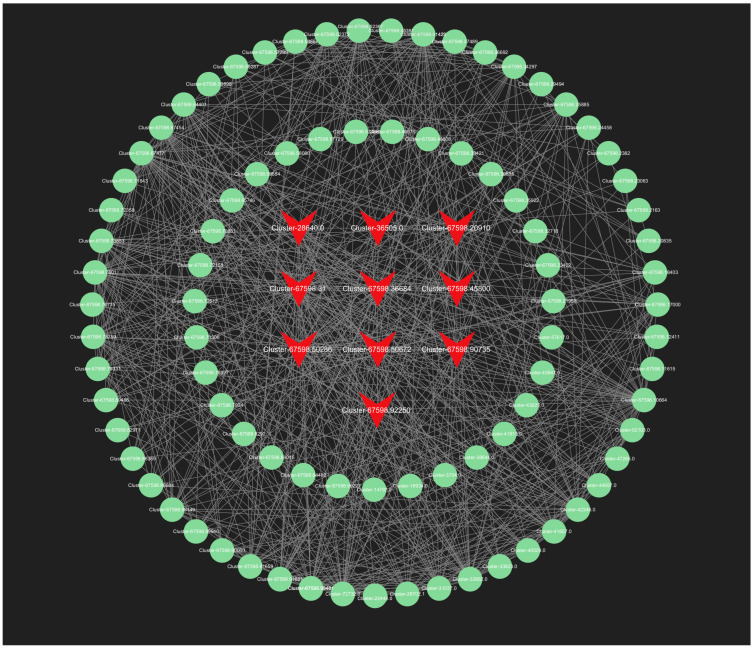
The all-gene networks in the orange module. The red color indicates the key transcriptional genes that have been screened.

**Figure 14:**
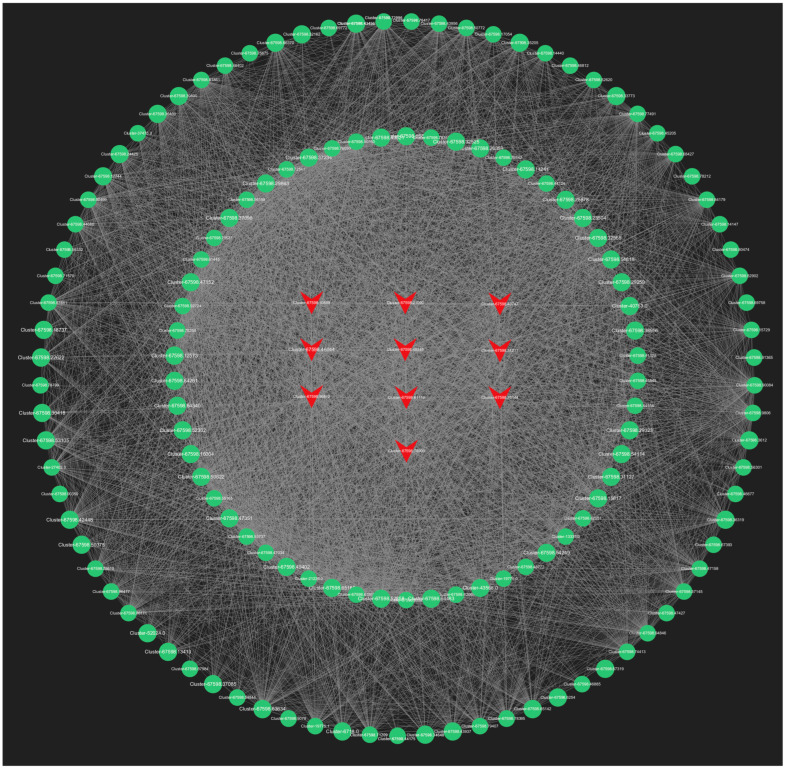
The top 150 connectivity gene networks in the light-yellow module. The red color indicates the key transcriptional genes that have been screened.

In our analysis, we screened all genes within the orange module, and the top 150 genes in the lightyellow module for connectivity to pinpoint the most critical genes associated with the response to root pruning. The results for the orange module, detailed in Figure 13, highlight the top 10 genes with the highest connectivity, strongly linked to the post-pruning response. These include *Cluster-67598.50286* (*TRZ1*), *Cluster-67598.20910* (*Polyubiquitin*), *Cluster-67598.36684* (*ACT11*), *Cluster-67598.64401* (*CCR4*), *Cluster-67598.92250* (*TMV resistance protein N*), *Cluster-67598.45800* (*ACT1*), *Cluster-36505.0* (*RPL37*), *Cluster-28640.0* (*RPS21*), *Cluster-67598.50672* (*O-fucosyltransferase 36-like*), and *Cluster-67598.90735* (*3-ketoacyl-acyl carrier protein synthase I*). The top 10 connectivity genes from the light-yellow module are presented in Figure 14 and include *Cluster-67598.56812* (*PGL3*), *Cluster-67598.48346* (*VPS4*), *Cluster-67598.54217* (*TLP1*), *Cluster-67598.46044* (*Kinase superfamily protein*), *Cluster-67598.75144* (*DSC1*), *Cluster-67598.61116* (*RD22*), *Cluster-67598.10589* (*receptor-like protein kinase haiku2*), *Cluster-67598.40747* (*PUP3*), *Cluster-67598.21090* (*GSO1*), and *Cluster-67598.78909* (*GLP4*). A detailed functional analysis of these 20 genes, as shown in Table S6, reveals their involvement in plant hormone signal transduction, growth, development, and stress resistance. Combined with the mechanism found above, *Polybiquitin*, *O-fucosyltransferase 36-like*, *VPS4*, *TLP1*, *RD22*, *PUP3*, and *GLP4* are closely related to the transport and expression of plant hormones, and it is speculated that these 7 genes may be the main reasons for the differences before and after treatment.

### 2.7. RT-qPCR Validation

**Figure 15:**
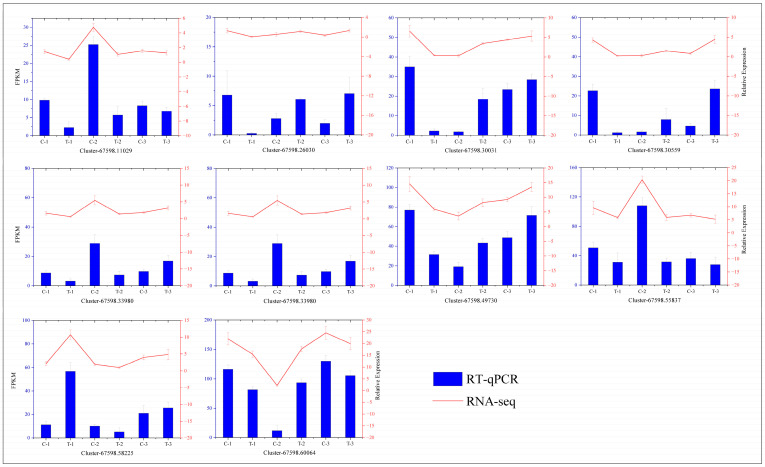
Correlation analysis between the RT-qPCR and RNA-seq results. The curve represents the RT-qPCR results, and the histogram displays the RNA-seq results. The leftmost axis shows the expression levels from transcriptome sequencing, and the rightmost axis represents the expression levels from RT-qPCR.

To verify the accuracy of our transcriptome data, we conducted RT-qPCR analyses on 10 differentially expressed genes selected at random and compared the results with those from the transcriptome expression levels. The outcomes, depicted in Figure 15, demonstrate consistent trends between the two methodologies, thus confirming the reliability of the transcriptome data.

## 3. Discussion

Research on root pruning in *Q. variabilis* is limited, with no comprehensive comparative analyses previously conducted between treated and control groups. This study utilized transcriptomic and hormone-profiling techniques to explore changes in *Q. variabilis* at various stages before and after root pruning. Our method generated 126.02 Gb of clean data and identified 7662 differentially expressed genes (DEGs), predominantly enriched in the plant hormone signal transduction pathway. Significant variances in hormone levels before and after pruning were observed by integrating hormone-profiling data, identifying these changes as the primary cause of phenotypic differences between the treated and control samples.

Root pruning mechanically injures the roots, triggering an immediate increase in defense-related hormones such as ABA and JA, while concurrently suppressing auxin secretion [33]. Studies, such as those by Marchant et al. [34], have shown that auxin promotes the development of lateral roots, indicating a positive regulatory role in their formation. Duan et al. [35] reported significant differences in hormone signaling between main and lateral roots of Arabidopsis, with salt stress inducing elevated ABA signaling specifically in lateral roots, underscoring ABA’s role in morphological regulation. Research by Busov [36] demonstrated that applying gibberellin (GA3) inhibits adventitious root formation in poplar stem cuttings, whereas Agusti et al. [37] observed that gibberellin enhances the proliferation of root meristem tissues, influencing lateral root development. These findings confirm that root pruning significantly affects endogenous hormone levels, which vary among saplings in response to such treatments. Consistent with the findings of this study, significant hormonal alterations post-pruning in *Q. variabilis* lead to increased lateral root formation compared to the control group.

In this research, we identified seven key genes, including *Polyubiquitin*, *O-fucosyltransferase 36-like*, *VPS4*, *TLP1*, *RD22*, *PUP3*, and *GLP4*, which likely account for the observed differences between the treatment and control groups. Further examination revealed that these genes are involved in hormone signal transduction or the growth and development of plant roots. For instance, Tang Y. H. [38] confirmed that CCR is a critical enzyme gene in the lignin synthesis pathway, playing a pivotal role in lignin metabolism and secondary metabolism. TPL proteins not only enhance plant tolerance to various abiotic stresses but also show high expression in specific plant tissues or developmental stages, notably during the ripening of fruits such as cherries, apples, and bananas. RD22, a member of the dehydration-induced RD gene family, is initially induced under drought stress within the ABA-signaling pathway [39,40,41,42]. Subsequent studies have expanded its role as a multi-factor-induced gene, responsive to low temperature, high salinity, and drought conditions [43,44,45,46,47]. The ABA response element (RYACGTGGYR) associated with RD22 also plays a role in drought induction and ABA response [48]. These findings further substantiate the significance of the identified key genes in this study.

## 4. Materials and Methods

### 4.1. Selection of Experimental Materials

The experimental site was located in the Third Residential Area of Henan Agricultural University in Zhengzhou City, which is situated in a subtropical humid monsoon climate zone. This region is characterized by mild weather and plentiful rainfall. The site averages about 2571 h of sunlight annually, with an average relative humidity of 69%, which fluctuates between a minimum of 17% and a maximum of 86%.

The experimental materials consisted of *Q. variabilis* seeds harvested from mature trees with uniform genetic backgrounds at the Huixian Forestry Farm in Xinxiang, Henan Province. Initially, the seeds were washed with clean water to remove soil and surface impurities. The selection criteria focused on seeds that were well-filled and uniform in shape, size, and color. These selected seeds underwent a sand burial germination process at an optimal temperature of 20 °C. Germination was considered successful once the seeds cracked open, revealing their white interiors. Subsequently, they were planted in nutrient pots measuring 12 cm × 10 cm × 9 cm, filled with a mix of sand, vermiculite, and perlite in a 3:1:1 ratio. Each pot contained one seed, which was adequately watered. Standard field management practices were maintained throughout the nursery phase, avoiding the use of pesticides or fertilizers. After the growth period, 180 similarly developed *Q. variabilis* saplings were selected for further experimentation. These saplings were uprooted, their root surfaces cleaned, and their main roots pruned to one-third of their original length to establish the treatment group, with unpruned saplings serving as controls. Each group, treatment, and control included three replicates of 30 saplings each. After pruning, the saplings were immediately replanted in nutrient pots.

On the 2nd day (new lateral root induction period), 12th day (new lateral root initiation and production period), and 25th day (new lateral root elongation period), 18 seedlings were collected from both the root-cutting group and the whole root control group, respectively (three seedlings per replicate in each group). The groups were labeled as T-1, T-2, T-3 for treatment and C-1, C-2, C-3 for control, according to the sampling period. The collected samples were wrapped in tin foil, rapidly frozen in liquid nitrogen, and stored at −80 °C for later analysis. Half of the samples were allocated for transcriptome sequencing, and the other half for metabolite testing. The remaining saplings were kept in ice boxes, ready for subsequent root morphology measurements, where three roots from each group (root-pruned and whole-root control) were selected to form a biological replicate.

### 4.2. Root Morphological Characteristics Measurement

For each treatment, three saplings with similar growth conditions were selected. Any sand or soil adhering to the roots was meticulously rinsed off under running water, and the roots were subsequently dried with absorbent paper. The root system was divided into two sections: the main root and the first-order lateral roots (L > 1 cm). The root samples were immediately scanned using an Epson Perfection V700 PHOTO scanner (Seiko Epson Corporation, Suwa, Nagano, Japan) at a resolution of 300 dpi. The scanned images were analyzed using WinRHIZO software (Version Pro 2007d, Régent Instruments Inc., Québec, QC, Canada) to determine total root length, root surface area, root volume, and the number of first-order lateral roots.

### 4.3. RNA Extraction, Library Construction, and Library Validation

RNA samples were extracted from various developmental stages of both the root-pruned and control groups using the Plant Polysaccharide Polyphenol RNA Rapid Extraction Kit (Tiangen, Beijing, China). RNA quality was ascertained through agarose gel electrophoresis and assessment with a NanoPhotometer spectrophotometer, which evaluated RNA integrity and checked for DNA contamination. Subsequently, ribosomal RNA was depleted to enrich for mRNA. The RNA was then fragmented using fragmentation buffer, which provided templates for synthesizing double-stranded cDNA. This cDNA underwent purification, end repair, A-tailing, and adapter ligation for sequencing. Size selection was performed with AMPure XP beads (Tiangen, Beijing, China), followed by PCR enrichment to construct the final cDNA library. Initial quantification of the library was conducted using a Qubit 2.0 Fluorometer, and insert size was verified using an Agilent 2100 Bioanalyzer (Agjilent, Santa Clara, CA, USA). After confirming the appropriate insert size, sequencing was performed on the Illumina Novaseq platform (Illumina, San Diego, CA, USA).

### 4.4. Sequencing-Data Processing

The cDNA libraries were sequenced on an Illumina HiSeq high-throughput sequencing platform. Sequencer-generated image data were transformed into raw data through CASAVA base calling. Initial data quality assurance was crucial to ensure the accuracy of downstream analyses. The fastp software version 0.19.3 [49] was employed for stringent quality control, checking sequencing error rates and the distribution of GC content in the fasta files. The high-quality reads, termed ‘clean reads’, were assembled using Trinity software version 2.15.1 [50]. The assembled transcripts were stored in FASTA format and served as reference sequences for further analyses. Hierarchical clustering of transcripts, based on read alignment and expression patterns, was conducted using Corset version 1.07 [51], selecting the longest sequence from each cluster as the Unigene for subsequent investigations.

### 4.5. Gene Function Annotation and Gene Expression Quantification

Unigene sequences were aligned against databases including the Kyoto Encyclopedia of Genes and Genomes (KEGG, https://www.genome.jp/kegg, accessed on 18 February 2023), NR, Swiss-Prot, Gene Ontology (GO, https://www.geneontology.org, accessed on 18 February 2023), COG/KOG, and Trembl using DIAMOND BLASTX software version 0.9.24.125 [52]. Predicted amino acid sequences of Unigenes were further aligned against the Pfam database using HMMER software version 3.3.2 to annotate the Unigenes. The deduplicated transcripts assembled by Trinity served as reference sequences for aligning clean reads from each sample using RSEM software version 1.3.1 [53], which quantified gene expression levels. Differential expression analysis was performed using DESeq2 version 1.22.2 [54], applying a threshold of |log2Fold Change| ≥ 1 and FDR < 0.05 to identify differentially expressed genes. Enrichment and annotation of these genes utilized the hypergeometric test along with resources from KEGG and GO.

### 4.6. Detection of Root Plant Hormone Profiling

The sample extracts were determined using an UPLC-ESI-MS/MS system equipped with column of Waters ACQUITY UPLC HSS T3 C18 (100 mm × 2.1 mm, 1.8 µm). The solvent system consisted of water with 0.04% acetic acid (A) and acetonitrile with 0.04% acetic acid (B). The gradient program for pump B started at 5% (0–1 min), then increased to 95% (1–8 min), continued to maintain at 95% (8–9 min), and finally ramped back to 5% (9.1–12 min). The flow rate was set to 0.35 mL/min, temperature was adjusted to 40 °C, and injection volume was set as 2 μL. Mass spectrometry was acquired in both positive and negative modes using electrospray ionization performed by Analyst 1.6.3 software (AB Sciex, Framingham, MA, USA). All phytohormones were analyzed via multiple reaction monitoring (MRM), and the mass spectrometry conditions were as follows: air curtain gas, 15 psi; spray voltage, 4500 V; nebulization gas pressure, 65 psi; auxiliary air pressure, 70 psi; and nebulization temperature, 400 °C. The selected reaction-monitoring conditions for protonated or deprotonated plant hormones ([M + H] or [M − H]^±^) are detailed in Table S7. Finally, Multiquant 3.0.3 software (Sciex, Framingham, MA, USA) was used for quantitative analysis.

Preparing 0.01 ng/mL, 0.05 ng/mL, 0.1 ng/mL, 0.5 ng/mL, 1 ng/mL, 5 ng/mL, 10 ng/mL, 50 ng/mL, 100 ng/mL, 200 ng/mL, and 500 ng/mL of standard solution with different concentrations (TRP and SAG are 20 times of the above concentration, that is, the standard curve concentration range is 0.2–10,000 ng/mL) obtained the mass spectrum peak intensity data of the corresponding quantitative signal of each concentration standard product. The concentration ratio between external standard and internal standard is the horizontal coordinate, the peak area ratio between external standard and internal standard is the longitudinal coordinate, and the standard curve of different substances is drawn. The ratio of the integrated peak area of all samples was calculated by substituting the linear equation of the standard curve and further substituting the calculation formula to obtain the content data of the substance in the actual sample.

The content of hormones in the sample (ng/g) = c × V/1000/m

(The meaning of each letter in the formula: c: the concentration value (ng/mL) obtained by substituting the ratio of the integrated peak area into the standard curve in the sample; V: the volume of the solution used in redissolution (μL); m: sample mass (g) taken).

The metabolite content data were normalized with a range method, and the accumulation patterns of metabolites in different samples were analyzed using R software version 4.4.1.

### 4.7. WGCNA

Before initiating the WGCNA, it is essential to preprocess the FPKM expression data by filtering out genes that show low expression across all samples, as well as those with consistent expression levels throughout the dataset. This preprocessing step is vital for enhancing the accuracy of network construction. The similarity in expression patterns between gene pairs is quantified by establishing a threshold for the correlation coefficient. A clustering dendrogram is then constructed based on the correlation among gene expression levels, from which modules are identified and selected for their optimal representation of gene interactions. These interactions are visualized by exporting the within-module data to Cytoscape V3.10.1 software. Genes that demonstrate strong interconnections are identified as hub genes and are functionally annotated by comparison with the Arabidopsis database (The Arabidopsis Information Resource, TAIR, https://www.arabidopsis.org, accessed on 23 February 2023).

### 4.8. Validation of DEGs with Real-Time Fluorescence Quantitative PCR (RT-qPCR)

RT-qPCR experiments were conducted using the same samples as those described previously. Total RNA extraction was performed first, followed by quality assessment, as outlined in Section 2.2. Reverse transcription was carried out using the HiScript II Q RT SuperMix for qPCR kit (Vazyme, Nanjing, China), which includes a genomic DNA removal step, strictly following the manufacturer’s protocol. Fluorescent quantitative primers were designed using NCBI-SmartBLAST, adhering to specific criteria: spanning exon-intron junctions, limiting product sizes to between 100 and 150 bp, maintaining primer Tm differences within 1 °C, and targeting a GC content of approximately 55% (refer to Table S1). The synthesized cDNA served as the template for qPCR, using *Q. variabilis QvActin* as the internal reference gene. A ChamQ Blue Universal SYBR qPCR Master Mix kit (Vazyme) was employed in a 20 µL reaction volume comprising: 10 µL of 2×SYBR qPCR Mix, 0.5 µL of each primer (10 µM), and 1 µL of cDNA. The PCR cycling conditions were as follows: initial denaturation at 95 °C for 2 min, followed by 40 cycles of 95 °C for 15 s, 60 °C for 30 s, and 72 °C for 30 s, concluding with a melting-curve analysis according to the instrument’s default settings. Each sample was tested in triplicate. Relative expression levels were quantified using a 2^−ΔΔCt^ method, and statistical significance was determined via multiple *t*-tests. The reliability and correlation between the transcriptome-sequencing results and the RT-qPCR data were confirmed, and the expression profiles of candidate genes implicated in rooting were evaluated using R packages to compare RT-qPCR with RNA-seq outcomes.

## 5. Conclusions

This study employed *Q. variabilis* as a model to examine the impacts of root pruning on the growth and development of the root system. Through transcriptomic analysis and hormone profiling, various hormones were identified that respond uniquely to root pruning in *Q. variabilis* saplings. Furthermore, bioinformatics tools helped identify seven critical genes that root pruning induces, significantly impacting the saplings’ root system development. The primary goal was to elucidate the molecular mechanisms by which root pruning influences root system development in these saplings. This research advances the theoretical frameworks within forest nursery practices and afforestation, providing a robust theoretical basis for developing scientifically supported vegetation restoration techniques.

## Data Availability

The basic data for this article can be found in this article. The transcriptome raw data have been uploaded to NCBI’s SRA database with Accession number: PRJNA1170239; the original data of hormone testing have been attached.

## Supplementary Materials

The supporting information can be downloaded at: https://www.mdpi.com/article/10.3390/ijms252111541/s1.
