# Supplementary material for: Research on the Molecular Mechanisms and Key Gene Discovery in Quercus variabilis Root Pruning Based on Transcriptomics and Hormone Profiling
Source: Int J Mol Sci. 2024 Oct 27;25(21):11541. doi: 10.3390/ijms252111541 (PMC11546583; doi:10.3390/ijms252111541)
Supplement: Supplementary file 1 [file ijms-25-11541-s001.zip › figure caption.pdf]

Figure S1: Sample Correlation Analysis. The numbers within the figure represent R2 values; values closer to 1 signify stronger correlations between the replicates.
Figure S2: Total ion flow chromatogram of metabolites;
Figure S3: Correlation diagram between different modules and samples.
Figure S4: Correlation clustering diagram of plant hormones across different samples. The horizontal axis denotes sample information, while the vertical axis pertains to metabolite information. The cluster tree on the left side of the diagram categorizes metabolite clustering. The color scale indicates expression levels post-normalization, with redder hues reflecting higher expression and gray indicating data not available (N/A). Group labels are provided for clarity
