# Supplementary figures and images for: Research on the Molecular Mechanisms and Key Gene Discovery in Quercus variabilis Root Pruning Based on Transcriptomics and Hormone Profiling
Source: Int J Mol Sci. 2024 Oct 27;25(21):11541. doi: 10.3390/ijms252111541 (PMC11546583; doi:10.3390/ijms252111541)

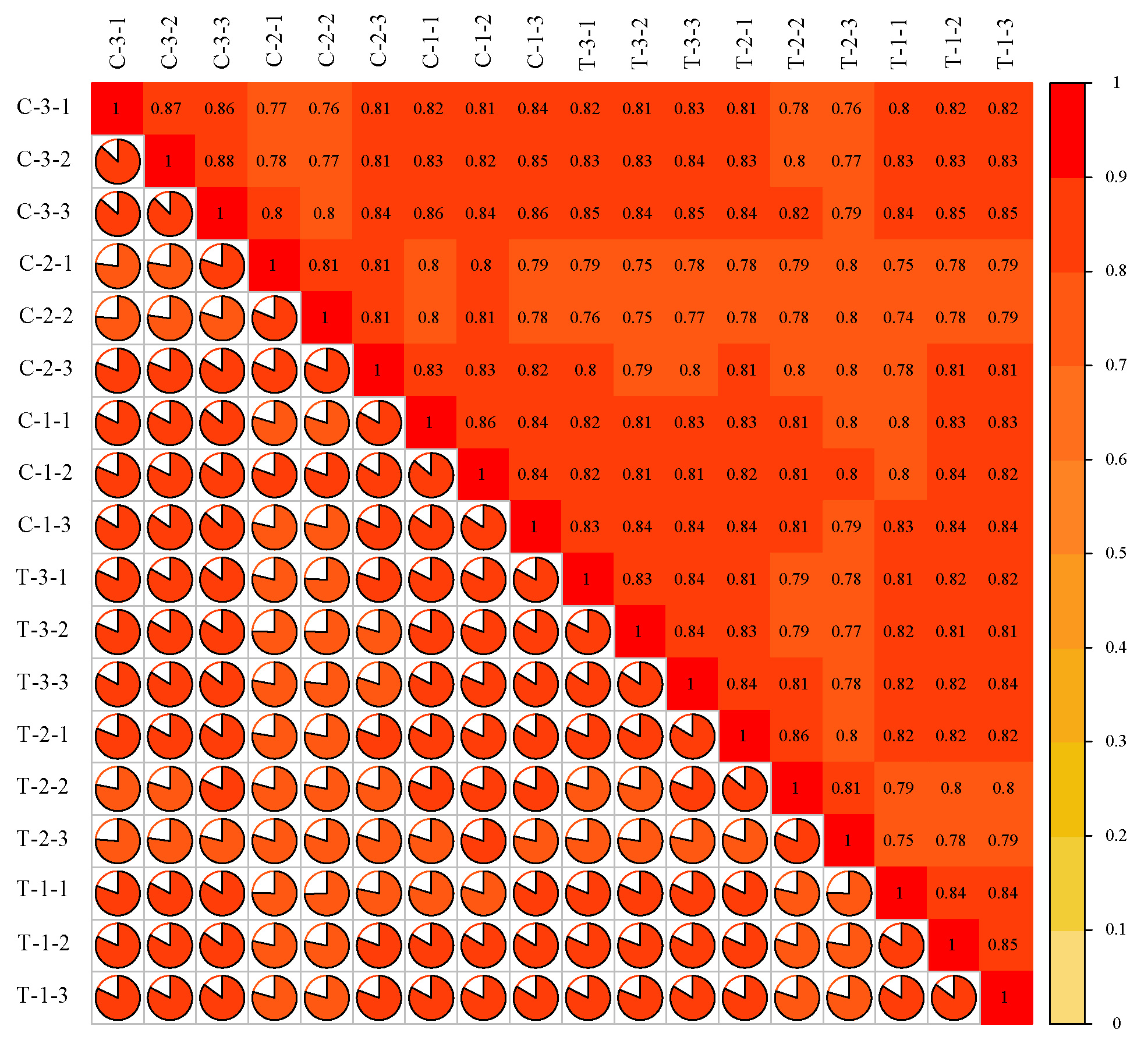

Supplement: Supplementary file 1 [file ijms-25-11541-s001.zip › Figure S1.jpg]

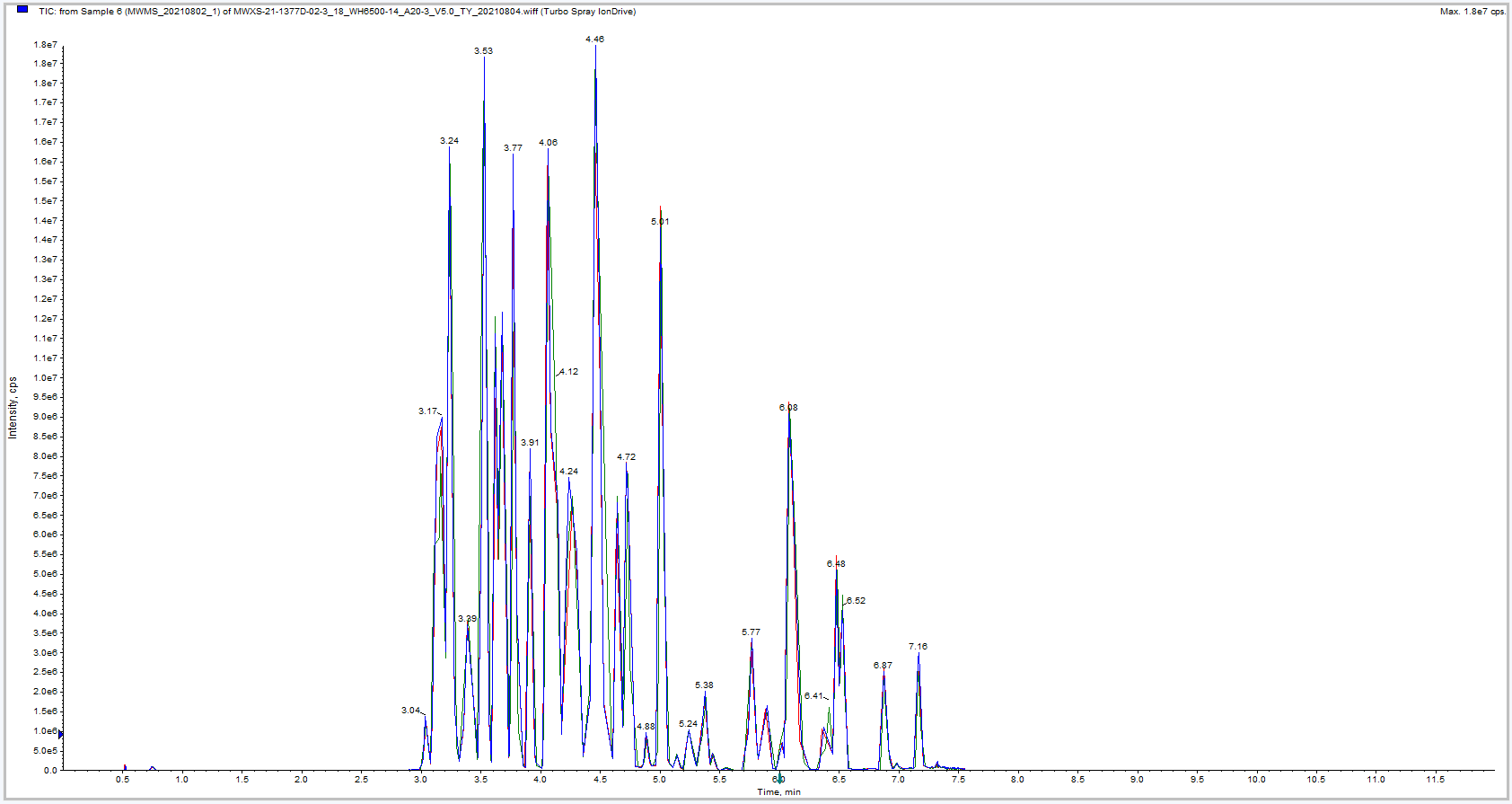

Supplement: Supplementary file 1 [file ijms-25-11541-s001.zip › Figure S2.png]

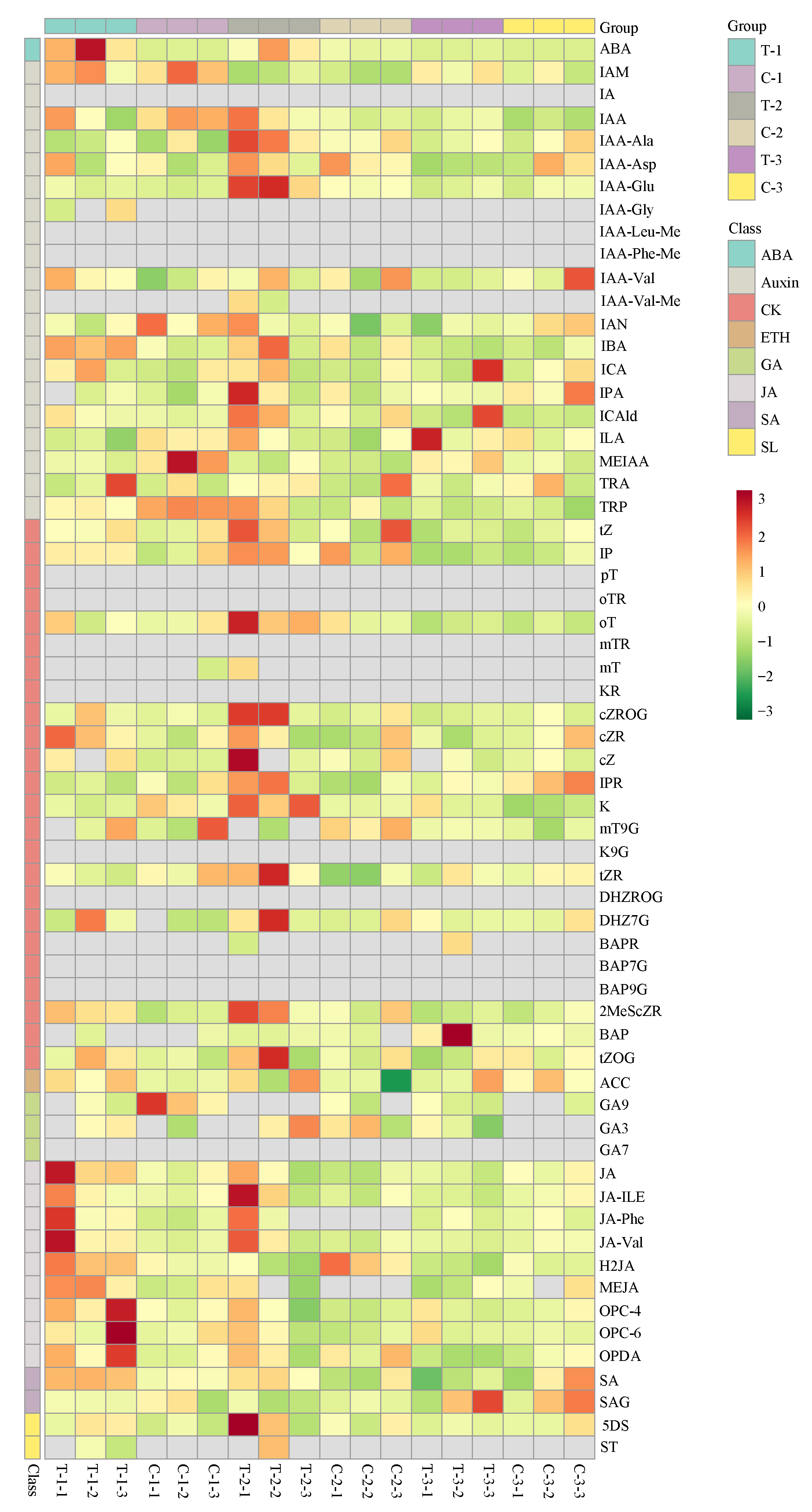

Supplement: Supplementary file 1 [file ijms-25-11541-s001.zip › Figure S3.jpg]

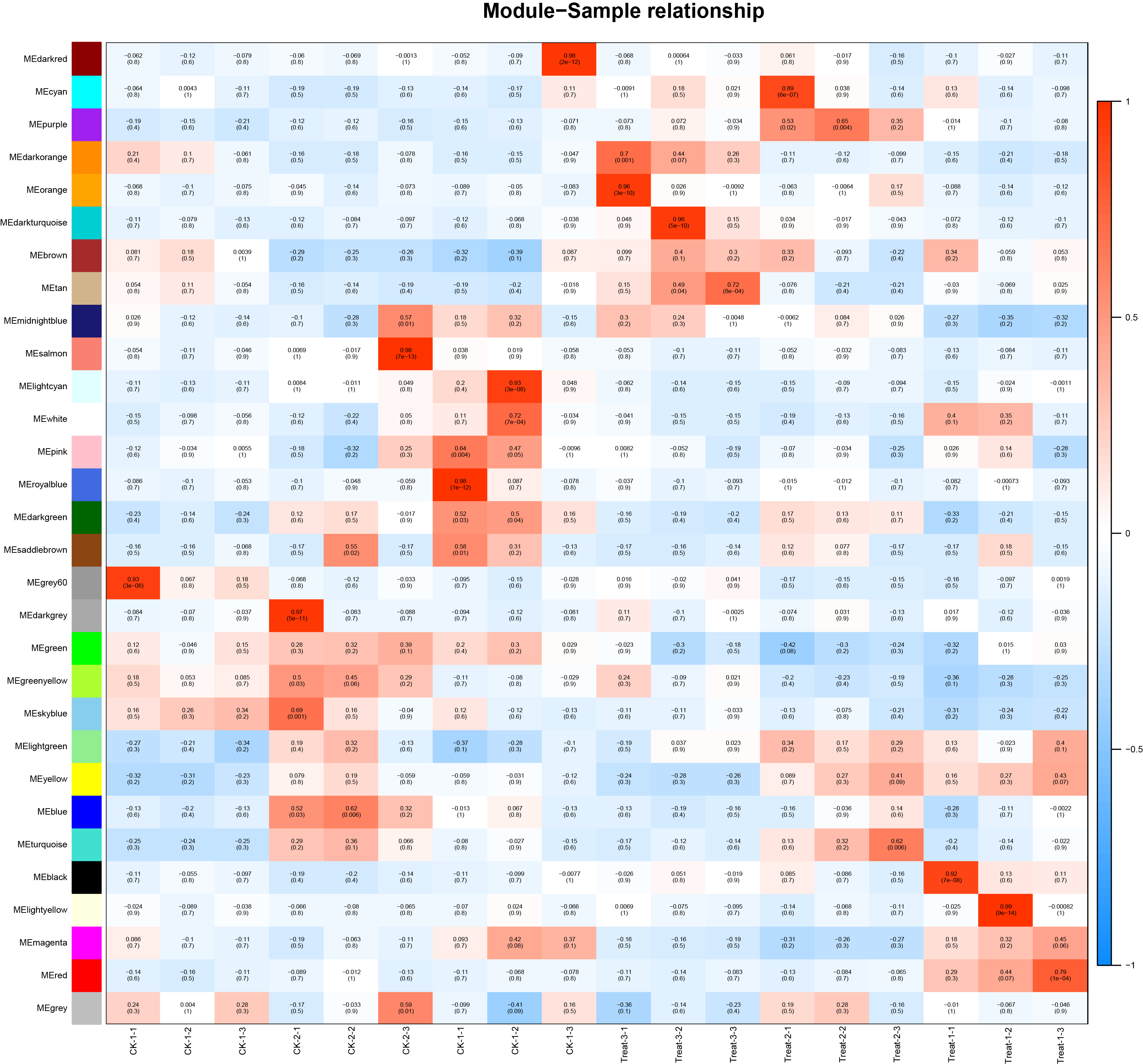

Supplement: Supplementary file 1 [file ijms-25-11541-s001.zip › Figure S4.jpg]
